# Editorial: The Neurodietetics and Genetics of Copper and Iron

**DOI:** 10.3389/fnmol.2021.722234

**Published:** 2021-07-23

**Authors:** Torben Moos, Lisbeth B. Møller

**Affiliations:** ^1^Neurobiology and Drug Delivery, Department of Health Science and Technology, Aalborg University, Aalborg, Denmark; ^2^Department of Clinical Genetics, Kennedy Center, Copenhagen University Hospital, Glostrup, Denmark

**Keywords:** copper, iron, blood-brain barrier, ATP7A, ATP7B, DNMT1

It is with great pleasure that we present this Research Topic dedicated to handling of transient metals and their function in the brain. Iron and copper are important co-factors for a number of enzymes in the brain, which are involved in multiple processes including neurotransmitter synthesis and myelin formation. Both shortage and excess of copper and iron will negatively influence brain function. The transport of copper and iron from peripheral circulation into the brain is strictly regulated and the concordantly protective blood-brain and blood-cerebrospinal fluid barriers have evolved to protect the brain's internal environment from unwanted exposure from the periphery. The sites for uptake and transport of copper and iron into the brain overlap, and the uptake mechanisms of the two metals significantly interact: Both iron deficiency and overload lead to altered copper homeostasis in the brain. Similarly, changes in dietary copper influences the cerebral iron homeostasis.

For long the focus of research on copper and iron in the brain has had a mainstay on the significance of molecules in the brain using these metals as co-factors. Moreover, the understanding of transport of the metals through the blood-brain and blood-cerebrospinal fluid barriers and the understanding of the further handling of the metals inside the brain have received prioritizing from many research groups. Naturally, the possibility of nutritional deficiency caused by insufficient dietary supply of copper and iron has also gained interest, and attempts to delineate the impact of dietary insufficient on the brain, not at least the developing brain, are important topics that could aid in understanding periods during development where sufficient metal supply could be a highest importance.

In 2015, we launched this Research Topic with the intention to provide a platform for presentation of research on the significance of copper and iron for the brain. This Research Topic mainly covers: (i) the genetics of proteins handling copper and iron in the brain, (ii) the expression and function of copper- and iron proteins in normal and pathological conditions; (iii) the clinic manifestations of dietary deficiencies in copper or iron; (iv) the clinic manifestation of genetic diseases leading to mishandling of copper and iron; (v) therapeutic aspects of handling dietary deficiencies, overloading pathologies, or conditions with genetic mutations in proteins related to copper and iron.

Møller et al. show that protein variants encoded by ATP7A transcripts missing either exon 10 or exon 15 are not functional and not responsible for the so-called occipital horn syndrome (OHS) phenotype, a mild variant of Menkes disease with mutation in the ATP7A gene. Prior studies have shown that only minor copper dependent transcriptional regulation of the ATP7A and ATP7B genes are apparent. In agreement with lack of rapid transcriptional regulation, the study of Lenartowicz et al. shows that a high copper concentration pressure leads to cellular selection. Interestingly, normal cells encoding the ATP7A gene have selective growth advantages at high copper concentrations, whereas cells without functional ATP7A has selective growth advantage toward low copper concentrations. The review by Lenartowicz et al. reports on the Mottle mouse, which closely recapitulates the phenotype of Menkes disease and leads to multi-systemic copper metabolism disorder caused by mutations in the X-linked ATP7A gene. Lessons from studies in the Mottled mouse reveal that the ATP7A protein is expelling copper from certain cells including cells in the kidney, intestine, placenta and testis, and that deficiency in ATP7A function leads to excessive or even toxic amounts of copper in these tissues and at the same time lack of copper in other tissues. Fu et al. show in their study on the role of copper in the developing ventricular system in the rat, that copper levels increase as a function of age, and subventricular zone shows a different expression pattern of Cu-regulatory genes than seen in the choroid plexus. An age-related increase in the copper-binding protein MTs and a simultaneous decrease in Ctr1 may contribute to the high copper level in this neurogenesis active brain region. The review of Skjørringe et al. discusses the role of the divalent metal transporter 1 (DMT1) for handling iron transport at the blood-brain barrier (BBB). DMT1 is detectable in endosomes of brain capillary endothelial cells denoting the BBB. Iron uptake at the BBB occurs by means of transferrin-receptor mediated endocytosis followed by detachment of iron from transferrin inside the acidic compartment of the endosome. McCarthy and Kosman discuss the transendothelial trafficking of iron at the BBB by covering mechanisms by which brain endothelial cells take-up iron from the blood and by which they efflux this iron into the abluminal space mediated by ferroportin. They also cover the regulation of iron efflux into the brain by exocrine factors released from adjacent astrocyte-end feet, and how cytokines secreted by the endothelial cells conversely may regulate such glial signaling. Codazzi et al. reviews the mechanisms responsible for non-transferrin-bound iron (NTBI) entry in neurons and astrocytes and on how they can be modulated during synaptic activity, not at least under the influence of calcium permeable channels and DMT1. They also in-depth speculate how NBTI might have relevance for cellular iron homeostasis in both physiological and pathological conditions. Gajowiak et al., studied changes in iron metabolism in a mouse model of amyotrophic lateral sclerosis (ALS). They report that overexpression of mutated SOD1^G93A^ leads to pathological changes in a skeletal muscle with deposits of iron.

This research forum hence covers many important characteristics of metal biology in the brain, and the advances thereof included within this Research Topic indeed brings novelty to the understanding of how the neurons and glial cells handles copper and iron to enable the significance of these essential metals for the brain.

## Author Contributions

LM and TM: summary of topic. Both authors contributed to the article and approved the submitted version.

## Conflict of Interest

The authors declare that the research was conducted in the absence of any commercial or financial relationships that could be construed as a potential conflict of interest.

## Publisher's Note

All claims expressed in this article are solely those of the authors and do not necessarily represent those of their affiliated organizations, or those of the publisher, the editors and the reviewers. Any product that may be evaluated in this article, or claim that may be made by its manufacturer, is not guaranteed or endorsed by the publisher.

